# Improvement in Grain Size Distribution Uniformity for Nuclear-Grade Austenitic Stainless Steel through Thermomechanical Treatment

**DOI:** 10.3390/ma17102313

**Published:** 2024-05-14

**Authors:** Yong Wang, Weiwei Xue, Zongxu Pang, Zichen Zhao, Zhuohua Liu, Chenyuan Liu, Fei Gao, Weijuan Li

**Affiliations:** 1 School of Materials and Metallurgy, University of Science and Technology Liaoning, Anshan 114051, China; agwangyong@163.com; 2 Iron & Steel Research Institutes of Ansteel Group Corporation, Anshan 114001, China; 3 Key Laboratory of Lightweight Structural Materials Liaoning Province, School of Materials Science and Engineering, Northeastern University, Shenyang 110819, China20224094@stu.neu.edu.cn (Z.Z.); 20224131@stu.neu.edu.cn (Z.L.); 20224353@stu.neu.edu.cn (C.L.);

**Keywords:** nuclear-grade austenitic stainless steel, microstructure uniformity, recrystallization nucleation, thermomechanical treatment, rolling reduction

## Abstract

In this work, thermomechanical treatment (single-pass rolling at 800 °C and solution treatment) was applied to nuclear-grade hot-rolled austenitic stainless steel to eliminate the mixed grain induced by the uneven hot-rolled microstructure. By employing high-temperature laser scanning confocal microscopy, microstructure evolution during solution treatment was observed in situ, and the effect of single-pass rolling reduction on it was investigated. In uneven hot-rolled microstructure, the millimeter-grade elongated grains (MEGs) possessed an extremely large size and a high Schmid factor for slip compared to the fine grains, which led to greater plastic deformation and increased dislocation density and deformation energy storage during single-pass rolling. During subsequent solution treatment, there were fewer nucleation sites for the new grain, and the grain boundary (GB) was the main nucleation site in MEGs at a lower rolling reduction. In contrast, at a higher reduction, increased uniformly distributed rolling deformation and more nucleation sites were developed in MEGs. As the reduction increased, the number of in-grain nucleation sites gradually exceeded that of GB nucleation sites, and in-grain nucleation preferentially occurred. This was beneficial for promoting the refinement of new recrystallized grains and a reduction in the size difference of new grains during recrystallization. The single-pass rolling reduction of 15–20% can effectively increase the nucleation sites and improve the uniformity of rolling deformation distribution in the MEGs, promote in-grain nucleation, and finally refine the abnormally coarse elongated grain, and eliminate the mixed-grain structure after solution treatment.

## 1. Introduction

Nuclear-grade austenitic stainless steel (ASS) can be used as a key structural material in the fusion reactor [1,2,3,4,5,6], such as 316LN-Mn, which was designed for the toroidal field coil structure in the China fusion reactor due to its extremely high strength, plasticity, and excellent toughness at cryogenic temperature. Compared to ferritic stainless steel, austenitic stainless steel was prone to dynamic recrystallization during hot rolling, which was conducive to microstructure refinement because it had a low tendency of dislocation cross-slip during hot rolling because of its low stacking fault energy and long distance between partial dislocations. However, during hot deformation, 316LN-Mn austenitic stainless steel tended to experience discontinuous dynamic recrystallization (DDRX) due to its lower stacking fault energy [7]. Furthermore, complete dynamic recrystallization (DRX) usually occurred at 1100 °C or above, and the temperature range that occurred at complete DRX was narrow. In this case, recrystallized grains were usually distributed in a ‘necklace structure’ around coarse, deformed grains, forming a kind of inhomogeneous rolled microstructure. According to our previous study [8], because adjacent grains possessed an uneven growth rate during the final solution treatment, this microstructure was very likely to cause mixed-grain structure in the final plate, which deteriorates the comprehensive performance of structural parts [9,10] and causes great potential safety hazards in actual production and life [11]. In the microstructure with smaller grains, the Cr-rich carbides are more prone to precipitation, increasing the intergranular corrosion susceptibility [12]. Therefore, in the mixed-grain structure, the region with fine grains preferentially undergoes intergranular corrosion, forming intergranular corrosion cracks, while in the microstructure with a uniform distribution of grain size, intergranular corrosion cracks are not easily formed.

Many researchers have paid attention to improving the mixed-grain structure, which can be attempted by several following approaches [13,14,15]: (1) Changing the chemical composition by adding a small amount of rare earth elements as inoculants to increase the nucleation sites. (2) Phase transformation. The austenite grain can be refined through controlled rolling and cooling, followed by phase transformation into ferrite, thereby achieving grain refinement in the ferrite phase. (3) Recrystallization. Changing the rolling and heat treatment parameters can adjust the recrystallization behavior (DRX or static recrystallization behavior) in different regions, finally improving the mixed-grain structure. Without altering the alloy composition and given that no phase transformation occurred during hot rolling [16], recrystallization is the sole method to improve the mixed-grain structure for nuclear-grade ASS.

Sui et al. [17] suggested that when using isothermal compression with a reduction rate of 50% at 1200 °C (that is, introducing DRX), the coarse grains in the mixed-grain structure are fully fragmented and refined. Yang et al. [18] found that for the 316 austenitic stainless steel heavy plate, as the total compression ratio was greater than 6, the homogeneous microstructure was obtained throughout the thickness, but when the pass reduction was less than 10%, the microstructure was heterogeneous even though the total compression ratio was high enough. However, these two approaches did not apply to 316LN-Mn austenitic stainless steel in this study because the mixed-grain microstructure after solution treatment was formed due to the narrow temperature range that occurred in complete DRX and the uneven microstructure that formed after hot rolling. In our previous study, during the solution treatment of the 316H stainless steel hot-rolled plate with an uneven microstructure, employing stepped heating contributed to the uniform growth of the grains during recrystallization and the development of the uniform distribution of grain size. However, this stepped heating required holding the solution treatment at different temperatures, which significantly diminished industrial production productivity. Although numerous achievements on austenitic stainless steel have been made in research regarding the relationship between microstructure and performance, most of them focus on microstructure development during cold and isothermal deformation [19,20,21], the establishment of a static recrystallization kinetic model during solution [22], the establishment of a crystal plasticity model of mixed-grain structure during the tensile process [23], the relationship between strength and grain size [24], and changing the chemical composition to improve grain size [25]. However, few studies have been carried out to improve microstructure uniformity based on hot rolling processes, especially for 316LN-Mn ASS.

For 316LN-Mn ASS in the present study, thermomechanical treatment (single-pass rolling at 800 °C and subsequent solution treatment) was introduced after hot rolling to eliminate the mixed-grain structure induced by the uneven hot-rolled microstructure. The change in uniformity of the grain size distribution after solution treatment with single-pass rolling reductions during thermomechanical treatment was studied. The strain partitioning on the uneven hot-rolled microstructure during single-pass rolling and its relationship with rolling reductions were analyzed, and through high-temperature laser scanning confocal microscope (HTLSCM), the microstructure evolution during final solution treatment, especially the new grain nucleation during the initial stage, was observed in situ and the influence of rolling reduction on this evolution was confirmed. Finally, the mechanism for improving microstructure uniformity was proposed, and the processing parameter for obtaining optimal microstructure uniformity was determined.

## 2. Materials and Methods

The experimental material received from Anshan Iron and Steel Group Co., Ltd., Anshan, China (Table 1) was a hot-rolled plate with a 30 mm thickness of 316LN-Mn austenitic stainless steel in this study, which possessed an inhomogeneous microstructure, as analyzed in Section 3.1. This initial hot-rolled plate was reheated to 800 °C and rolled in a single pass with different reductions (7%, 10%, 13%, 15%, and 20%), and finally subjected to solution treatment at 1100 °C for 33 min. The final plates with different single-pass rolling reductions were obtained.

In situ observation during solution treatment was conducted on a VL2000DX-SVF17SP&15FTC HTLSCM. Before the in situ observation, the cylindrical sample with the size of Φ6.5 mm × 3.5 mm was placed in the sample chamber of HTLSCM after grinding and mechanical polishing, and multiple vacuuming cycles were operated to avoid oxidation at high temperatures. During the in situ observation, the sample was heated to 1100 °C at 20 °C/s and held for 3 s or 300 s, and then rapidly cooled to room temperature at 100 °C/s using high-purity helium. A long holding time (300 s) was used to observe microstructure evolution within the entire observation area during solution treatment, while a short holding time (0 s) was used to observe the grain boundary (GB) migration and new grain nucleation within the local area during the early stage of solution treatment. Note that in the literature [26,27], the principle of observing GB during high temperatures using HTLSCM has been introduced, and its corresponding reliability has been proved. The microstructure of the sample was collected at a frequency of 0.1 Hz during the in situ observation experiment of solution treatment.

Microstructure analyses were conducted on an Olympus BX53M optical microscope. The corresponding sample was ground, mechanically polished, and subsequently chemically etched. Chemical etching was performed in a solution containing 5 g of ferric chloride, 20 milliliters of water, and 20 milliliters of hydrochloric acid. EBSD analyses were performed utilizing the Oxford EBSD detector (model: Symmetry S2) mounted on a ZEISS Crossbeam 550 dual-beam focused ion beam-scanning electron microscope. The sample for EBSD analysis was ground, ultrasonically cleaned, and finally electrolytically polished to release the stress. Electrolytic polishing was performed in a solution containing 650 milliliters of alcohol and 100 milliliters of perchloric acid, and its parameters included an electrolytic voltage of 25 V, electrolytic time of 25 s, and electrolytic current of 0.5–1.5 A. The EBSD measurement parameters are the following: working distance of 12.5 mm, accelerating voltage of 20 kV, and step size of 1.5–2 μm. The use of AZtecCrystal 2.2 software processed the data obtained from the EBSD measurement. The Vickers hardness test was conducted on a KB3000BVRZ-SA macroscopic universal hardness tester under the test force of 20 Kg. The preparation of the corresponding sample was consistent with that for microstructure analyses. Note that the hardness at each condition was the average of the three measured values.

## 3. Results and Discussion

### 3.1. Microstructure after Single-Pass Rolling with Different Reductions

**Figure 1:**
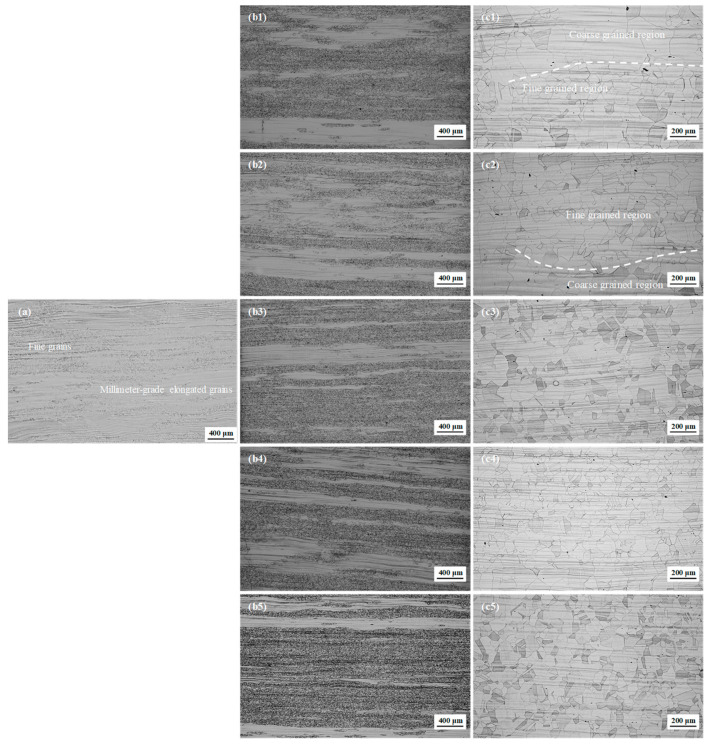
Initial hot-rolled microstructure (**a**) and microstructures after subsequent single-pass rolling (**b1**–**b5**) and after final solution treatment with different reductions (**c1**–**c5**); (**b1**,**c1**) 7%, (**b2**,**c2**) 10%, (**b3**,**c3**) 13%, (**b4**,**c4**) 15%, and (**b5**,**c5**) 20%.

Figure 1 displays the microstructure evolution with single-pass rolling reductions of 7%, 10%, 13%, 15% and 20%. The microstructure of the initial hot-rolled plate exhibited very fine grains (FGs) and millimeter-grade elongated grains (MEGs); that is, the hot-rolled microstructure was inhomogeneous. This indicates that during hot rolling, partial dynamic recrystallization took place. Based on our previous study [8], if this inhomogeneity hot-rolled microstructure is directly subjected to solution treatment, it is highly likely to induce a mixed-grain structure, as shown in Figure S1. In this study, this microstructure in the initial hot-rolled plate subsequently underwent single-pass rolling at 800 ℃ and corresponding solution treatment. After single-pass rolling, a prominently inhomogeneous microstructure was still observed for different reductions, such as some MEGs and very fine grains. The average width of the MEGs decreased distinctly with the increase in single-pass rolling reduction, while no significant changes in the FGs were observed. Note that there are some differences in the number of MEGs in Figure 1(b4,b5). This might be due to the random occurrence of DRX in the microstructure during hot rolling, resulting in the uneven distribution of MEGs in the initial hot-rolled plate. After the final solution treatment, new equiaxed grains developed in all microstructures. However, the grain size distribution was significantly different for the final plates with different rolling reductions. Under lower reductions (below 15%), there was a large difference in grain size, and the inhomogeneous microstructure was inherited into the final plates, and a mixed-grain structure was formed, as shown in Figure 1(c1–c3). At higher reductions (above 15%), the size difference between larger and smaller grains was reduced, the distribution of grain size in the final plates tended to be even gradually, and no mixed-grain structure was observed, as shown in Figure 1(c4,c5). The uniformity of the microstructure in the final plate continuously increased with single-pass rolling reduction, and the uniform distribution of the grain size was formed after 15% and 20% reductions.

**Figure 2:**
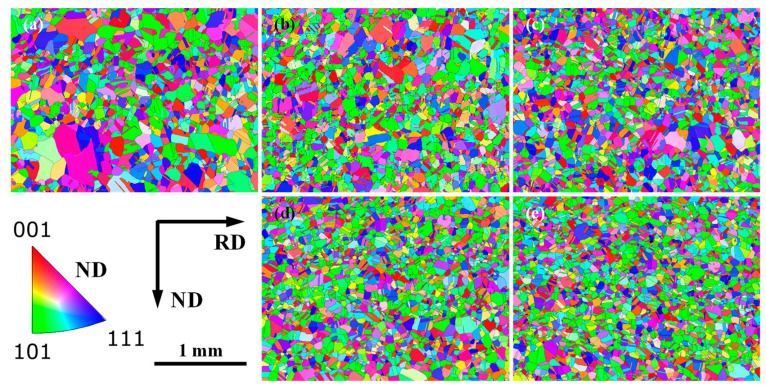
EBSD analysis of microstructures after solution treatment under different rolling reductions: (**a**) 7%, (**b**) 10%, (**c**) 13%, (**d**) 15%, and (**e**) 20%.

**Figure 3:**
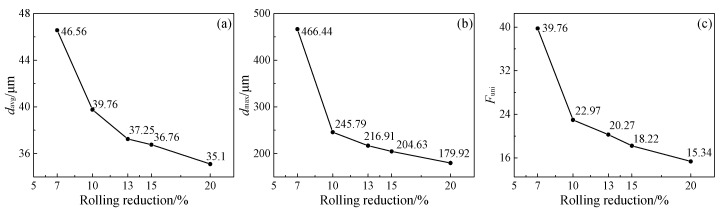
Evaluation of microstructure uniformity after solution treatment under different rolling reductions: (**a**) *d_avg_*, (**b**) *d_max_*, and (**c**) *F_uni_*.

To quantitatively evaluate the uniformity of the grain size distribution, the microstructure after the final solution treatment was studied using EBSD, as shown in Figure 2. In this study, the uniformity factor of grain size distribution *F_uni_*, the maximum grain size *d_max_*, and the average grain size *d_avg_* were employed for evaluating the grain size distribution uniformity or microstructure uniformity. Note that *F_uni_* was determined by the ratio of *d_max_* to *d*_0_ (*d*_0_ is the grain size that occurs most frequently.). Taking into account the presence of a mixed-grain structure, the different maximum grain sizes *d_max_* were obtained in different analyzed areas for the final plate. To decrease this difference, the large area (1800 μm × 1334 μm) for the EBSD measurement was employed, and *d_max_* was determined by the average size of three larger grains in this analyzed area. Undoubtedly, lower *d_avg_*, *d_max_*, and *F_uni_* indicated the higher uniformity of grain size distribution and less pronounced mixed-grain structure in the microstructure. These parameters for different single-pass rolling reductions were determined by EBSD analysis (Figure 2) and are presented in Figure 3. With the increase in reduction, these parameters of *d_avg_*, *d_max_*, and *F_uni_* decreased. This indicated that the greater the rolling reduction, the less clear the mixed-grain structure and the greater the microstructure uniformity. The microstructure observation and corresponding quantitative analysis above revealed that when the uniformity factor of the grain size distribution *F_uni_* reached below 18.2 at the single-pass rolling reduction of 15% above, the grain size distribution became more uniform, and the mixed-grain structure disappeared. Introducing single-pass rolling at 800 °C with a higher reduction (above 15%) can improve the microstructure uniformity and eliminate the mixed-grain structure for experimental steel. This indicated that, in addition to the applications of thermomechanical treatment analyzed by Lucchese et al. [28], the thermomechanical treatment can also eliminate the mixed-grain structure.

### 3.2. Microstructure Evolution during Solution Treatment by In-Situ Observation

**Figure 4:**
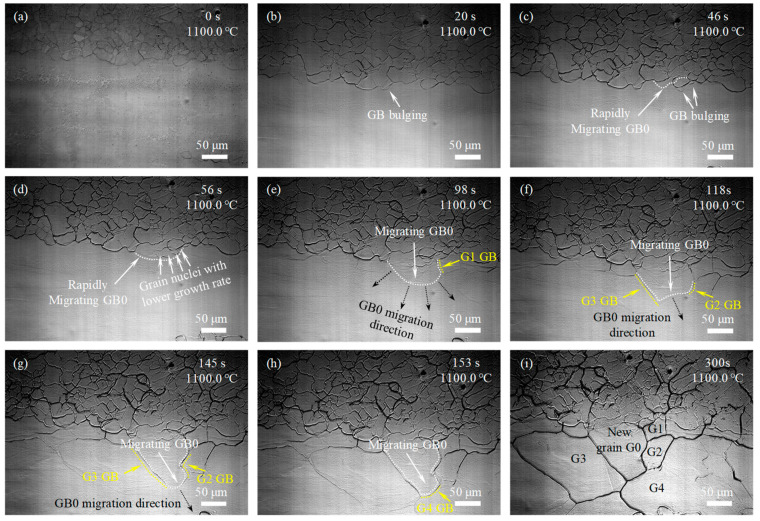
In situ observation of microstructure development during solution treatment at 1100 °C for the experimental steel with 7% reduction: (**a**) 0 s; (**b**) 20 s; (**c**) 46 s; (**d**) 56 s; (**e**) 98 s; (**f**) 118 s; (**g**) 145 s; (**h**) 153 s; and (**i**) 300 s.

Figure 4 shows the in situ observation of microstructure development during solution treatment for the experimental steel with 7% single-pass rolling reduction, and Figure 4a–i shows the different microstructures in the same region during solution treatment at different times. As the temperature reached 1100 °C for 20 s, GB migration began to occur in the GB of MEGs through GB bulging to the interior of the MEGs, as labeled in Figure 4b, and new grain nuclei began to form gradually. With the increase in the holding time, greater GB bulging developed at the GBs of MEGs, as marked in Figure 4c, and some GBs began to migrate rapidly as the recrystallization area extended towards the deformed microstructure in MEGs, like the rising tide. Finally, the deformed microstructure completely transformed into new recrystallized grains, and many new grains developed. To provide a comprehensive understanding of new grain formation, the microstructure evolution was thoroughly analyzed, and the typical area was depicted as follows.

At the soaking time of 46 s, GB GB0 (indicated by the long arrows in Figure 4c) began to migrate rapidly. This GB consumed some previously formed grain nuclei with a lower growth rate (indicated by the short arrows in Figure 4d) during its migration. Subsequently, it continued to migrate towards the surrounding deformed area, but the migration rate decreased. During continuous migration, the GB GB0 met with the GB of surrounding newly formed grains (such as G1 GB in Figure 4e, G2 GB and G3 GB in Figure 4f,g, and G4 GB in Figure 4h), forming ‘collision front lines’ and ultimately stopping migration and forming new GBs. Finally, a new grain G0 was developed, and its GB consisted of the newly formed GBs mentioned above (Figure 4i). These results indicated that during recrystallization, the formation of new GBs in the MEGs was achieved primarily through the collision and joining of GBs after their bulging and migration, and the nucleation of new grains was mainly concentrated at GBs.

**Figure 5:**
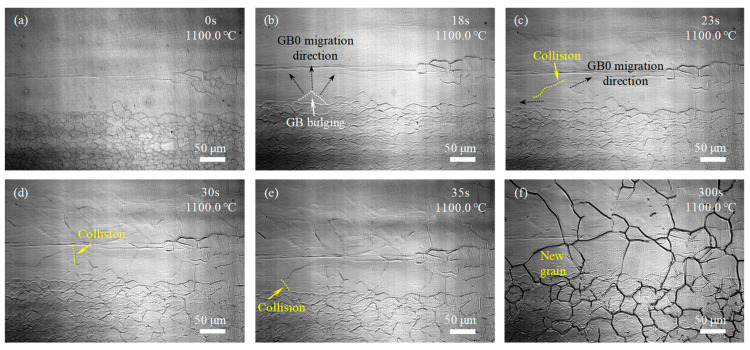
In situ observation of microstructure development during solution treatment at 1100 °C for the experimental steel with 10% reduction: (**a**) 0 s; (**b**) 18 s; (**c**) 23 s; (**d**) 30 s; (**e**) 35 s; and (**f**) 300 s.

**Figure 6:**
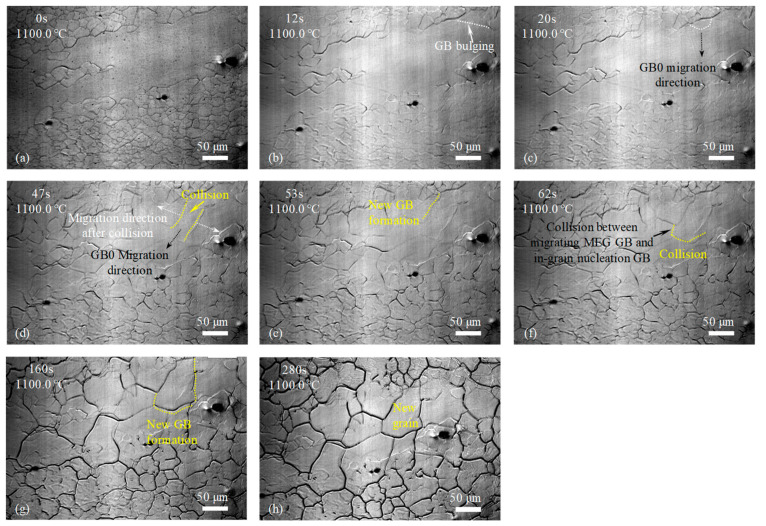
In situ observation of microstructure development during solution treatment at 1100 °C for the experimental steel with 15% reduction: (**a**) 0 s; (**b**) 12 s; (**c**) 20 s; (**d**) 47 s; (**e**) 53 s; (**f**) 62 s; (**g**) 160 s; and (**h**) 280 s.

**Figure 7:**
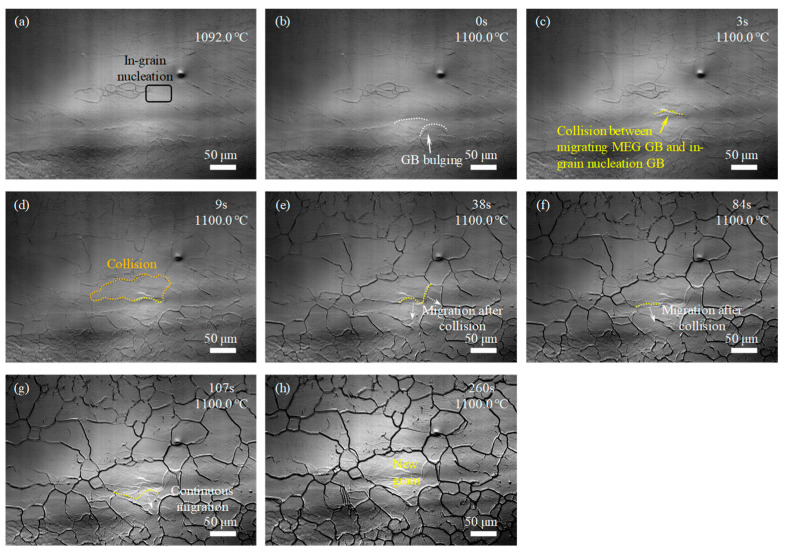
In situ observation of microstructure development during solution treatment at 1100 °C for the experimental steel with 20% reduction: (**a**) 0 s; (**b**) 20 s; (**c**) 46 s; (**d**) 56 s; (**e**) 98 s; (**f**) 118 s; (**g**) 145 s; (**h**) 153 s.

For the samples with the single-pass rolling reductions of 10%, 15%, and 20%, the GBs of MEGs began to migrate through GB bulging or newly formed GB in the interior of MEGs began to migrate, and new grains began to form after holding at 1100 °C for 18 s and 12 s and before heating to 1100 °C, as labeled in Figure 5b, Figure 6b and Figure 7a, respectively. The incubation time of recrystallized grain nucleation decreased with increasing rolling reduction during the final solution treatment. This manifested that the rolling deformation has a prominent impact on the incubation of recrystallized grain nucleation. Moreover, as the holding time was prolonged, more MEGs with GB bulging occurred, more newly formed GBs appeared in the interior of MEGs, and more GBs began to migrate; eventually, the MEGs were completely replaced by many new nucleated grains, which was similar to the recrystallization of the sample with rolling reductions of 7%.

The microstructure evolution during the solution treatment for samples with rolling reductions of 10%, 15%, and 20% (Figure 5, Figure 6 and Figure 7) were also analyzed by the approaches used in Figure 4c–h. It was found that at lower rolling reductions (such as 10%), the new GBs in the MEGs during recrystallization developed via the bulging and migration of MEG GBs and the collision and joining of migrating GBs; that is, the nucleation of new grains in the MEGs mainly relied on the MEG GBs. By contrast, at higher rolling reductions (such as 15% and 20%), besides the above methods, many new GBs were also formed by the collision and joining between the migrating MEG GBs and the migrating GBs developed at the nucleation sites of new grains within MEGs. The new grains in the MEGs nucleated at the MEG GBs and within the MEGs during recrystallization; that is, both GB nucleation and in-grain nucleation occurred together. Moreover, at a rolling reduction of 20%, the in-grain nucleation occurred before the GB nucleation.

In addition to the differences in the nucleation incubation period and site above, new recrystallized grains that replaced the deformed microstructures exhibited a difference in size for samples after different rolling reductions. Increasing the rolling reduction was conducive to the new grain refinement, which could be attributed to the increase in the nucleation sites. In order to quantitatively analyze these differences, the number of GB and in-grain nucleation sites in MEGs during recrystallization were analyzed based on the in situ observation above (Figure 4, Figure 5, Figure 6 and Figure 7), as shown in Table 2. Clearly, the nucleation site and its number displayed significant differences in the samples after different rolling reductions. The number of nucleation sites increased with rolling reduction, and this change was more significant at a higher reduction.

Furthermore, in the same sample, new recrystallized grains that developed in deformed microstructures possessed significant size differences, especially in samples with lower deformation amounts. Generally, the inhomogeneous distribution of deformation in MEGs was much clearer during single-pass rolling with lower reductions. This resulted in an uneven distribution of dislocation density and deformation storage energy, inducing the different recrystallization behaviors (such as the recrystallization driving force and the number of nucleation sites) of different areas in MEGs. Finally, the size difference of new grains formed in different areas. These results indicated that a higher single-pass rolling reduction was beneficial for promoting the refinement of new recrystallized grains and lowering the difference in the size of the new grains. Undoubtedly, the refinement of new recrystallized grains and the homogenization of their size distribution were conducive to the improvement of microstructure uniformity and the elimination of mixed-grain structure after solution treatment.

### 3.3. Microstructure Uniformity Improvement Mechanism

**Figure 8:**
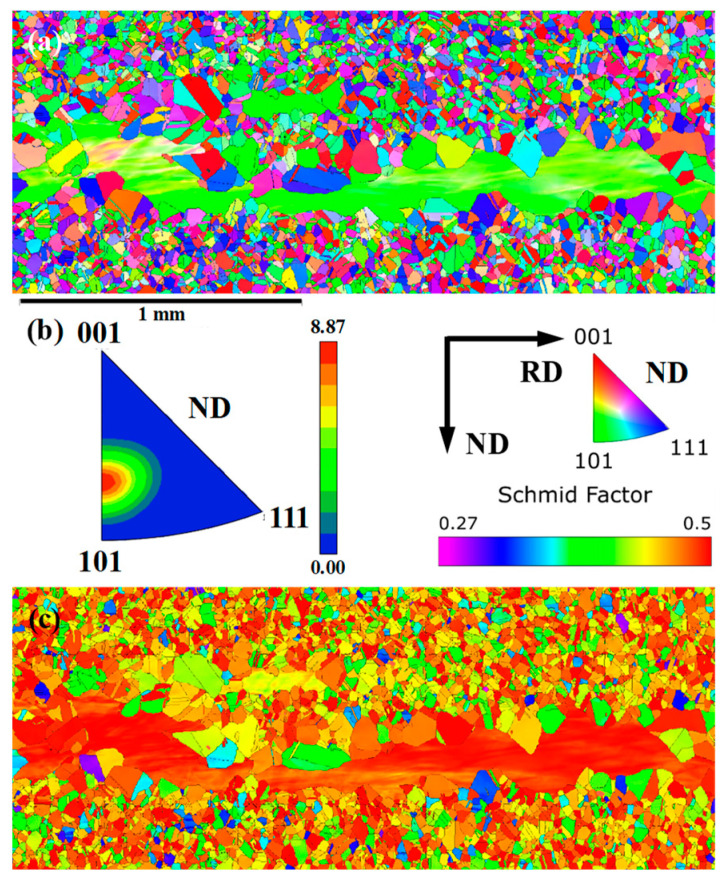
EBSD analysis of the initial hot-rolled microstructure; (**a**) Distribution of all orientations; (**b**) Texture of MEGs in microstructure; (**c**) Schmidt factor distribution. (Textures were exhibited by using the inverse pole figure).

Through the in situ observation of microstructure development during solution treatment, it was pretty clear that for experimental steel with different single-pass rolling reductions, no significant differences in the microstructures within the area with FGs were observed during solution treatment, while the microstructure in the MEGs displayed a striking difference in grain size and its distribution. This indicated that single-pass rolling had a significant impact on the recrystallization behavior in the area of MEGs, but its effect on the area of FGs was not clear. In order to deeply analyze this phenomenon, the microstructure of the initial hot-rolled plate was studied using EBSD (Figure 8) and the hardness test.

In the microstructure of the initial hot-rolled plate, there were clear MEGs due to partial DRX during hot rolling, as shown in Figure 8a. This indicated that these MEGs experienced hot rolling deformation below the austenite recrystallization temperature. Moreover, these MEGs displayed strong texture, as shown in Figure 8b. A similar relationship between austenite deformation and texture has been observed and proved by many researchers [29]. Extensive work found that after hot rolling at a temperature lower than the recrystallization temperature of austenite, a sharp texture was developed for deformed austenite, containing S {123}<634>, brass {110}<112>, copper {112}<111>, and weak {110}<001> components [29]. Furthermore, numerous studies found that polycrystal deformation was closely related to grain orientation. Meng et al. [30] calculated the Schmid factor of austenite for twinning and slip during deformation and plotted the Schmid factor contours for twinning and slip in the orientation triangle, corresponding to the results reported by Yu [31]. Based on the Schmid factor distribution reported by Meng et al. [30] and Yu [31], the texture components of MEGs in the initial hot-rolled microstructure were concentrated near the orientation with the highest Schmid factor for slip during deformation. Hence, MEGs possessed a higher Schmid factor for slip than FGs during deformation. This was consistent with the calculated Schmidt factor distribution for the initial hot-rolled microstructure based on EBSD analysis (Figure 8c). These results indicate that, during deformation, MEGs tend to preferentially undergo plastic deformation compared to FGs.

In addition, Vickers hardness values in the regions with MEGs were 203.7 ± 4.5, while those with FGs had a value of 219.1 ± 5.2, indicating that microstructures with MEGs usually exhibited a lower hardness than those with FGs. Hardness represents the ability of a material to locally resist hard objects that press onto its surface. Generally, during rolling, the stress state is characterized by tensile stress in one direction and compressive stress in two directions, and the contact surface between the roll and the experimental steel is subjected to compressive stress. Under this stress state, the regions with lower hardness in the experimental steel had weaker resistance to deformation and were more prone to deformation during rolling deformation. Based on the orientation analysis and hardness test, during subsequent deformation, the regions with MEGs in the initial hot-rolled microstructure experienced deformation preferentially compared to those with FGs; that is, the regions with MEGs found it easier to undertake larger plastic deformation and accumulate higher dislocation density and deformation energy storage during single-pass rolling, which can influence the recrystallization behavior during solution treatment. In this study, single-pass rolling can produce a more remarkable influence on recrystallization behavior in regions with MEGs than those with FGs.

**Figure 9:**
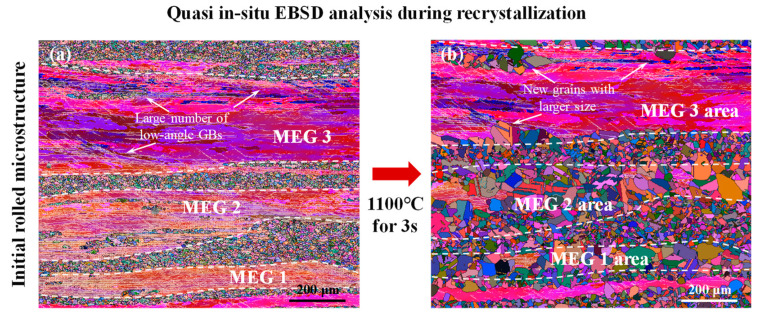
Microstructure evolution during initial stage of recrystallization for the sample with 20% rolling reduction studied by quasi in situ EBSD analysis: (**a**) Euler map of initial rolled microstructure; (**b**) Euler map of microstructure after holding at 1100 °C for 3 s.

For in-depth analysis of the change in microstructure evolution during solution treatment for experimental steel with different single-pass rolling reductions, the microstructure evolution during recrystallization of the sample after 20% reduction was analyzed by quasi in situ EBSD analysis, as shown in Figure 9. This quasi in situ study was achieved by combining the HTLSCM experiment and EBSD measurement as follows: after EBSD analysis, the initial hot-rolled sample was heated to 1100 °C at 20 °C/s and held for 3 s in the HTLSCM and then rapidly cooled to room temperature at 100 °C/s, and finally, this sample during partial recrystallization was analyzed again by EBSD. For the sample with a reduction of 20%, many low-angle GBs were observed in MEGs, as shown in Figure 9a, which further confirmed that during single-pass rolling, MEGs tended to undertake larger plastic deformation. These low-angle GBs were mainly distributed near MEG GBs and in local areas within MEGs. Furthermore, in MEGs 1 and 2, the number of low-angle GBs was relatively large, while MEG 3 possessed fewer low-angle GBs. This could be due to the presence of heterogeneous deformation during single-pass rolling.

After being kept at 1100 °C for 3 s, most areas of MEGs 1 and 2 were consumed by new recrystallized grains, as shown in Figure 9b, and these new grains were developed at the GBs and the interior of the MEGs; that is, both in-grain nucleation and GB nucleation occurred. This also demonstrated that after a higher rolling reduction, the nucleation incubation time was significantly shorter than that at a lower reduction. Nevertheless, in MEG 3, only part of MEG 3 was consumed by new nucleated grains, and the new grains with larger sizes were developed in the area with many low-angle GBs, as shown in Figure 9b. The number of new grains in MEG 3 was relatively small compared to MEGs 1 and 2, as shown in Figure 9b. During single-pass rolling at 800 °C, experimental steel tended to occur with dynamic recovery during deformation. Lattice defects (such as dislocations) were annihilated and rearranged, inducing low-angle GBs. The formation of the low-angle GBs was compactly connected with dislocations. The regions with many low-angle GBs possessed high dislocation density and deformation storage energy, which are conducive to enhancing the nucleation sites, promoting grain growth, and heightening the driving force for recrystallization during solution treatment [32]. Hence, the number of new grains in MEGs with more low-angle GBs was higher, and their size in the local area of MEGs with a greater number of low-angle GBs was larger. There was a close correlation between the new grain nucleation during solution treatment and the dislocation activity in the corresponding slip system during rolling deformation.

**Figure 10:**
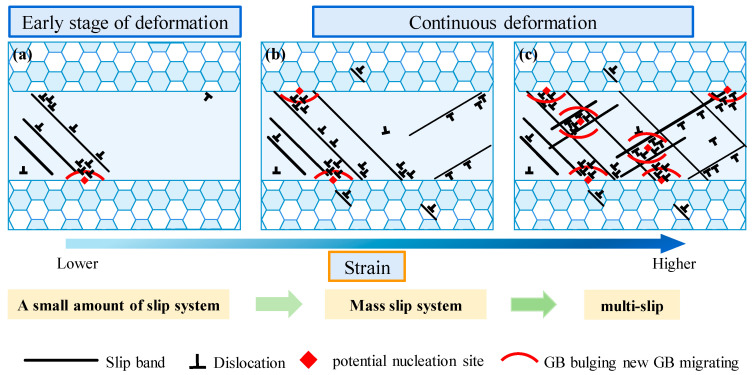
Schematic diagram of microstructure uniformity improvement mechanism: (**a**) early stage of deformation; (**b**) lower strain; and (**c**) higher strain.

For the initial hot-rolled microstructure, the regions with MEGs and FGs experienced varying degrees of plastic deformation during single-pass rolling and had different dislocation distributions and deformation energy storage, thereby affecting the recrystallization behavior of different regions during final solution treatment. These differences varied with the change in the single-pass rolling condition and finally resulted in a variation in the uniformity of the grain size distribution. The recrystallization behavior under different deformation conditions and the improvement mechanism of microstructure uniformity were further elaborated, as shown in Figure 10. Figure 10a–c shows the schematic diagram of microstructure at different strains and its effect on recrystallization behavior.

During single-pass rolling, the strain partitioning was uneven in the initial microstructure with MEGs and FGs due to the different deformation tendencies. At the early stage of single-pass rolling deformation, only the primary slip system was activated in the initial microstructure, and the corresponding dislocation activity occurred in this slip system, resulting in the development of the slip band. These slip bands were arranged in one direction. Undoubtedly, GBs can obstruct slip band development and make dislocation activity difficult, leading to dislocation pileups and high dislocation density in GBs. Thus, the local location of the GBs became potential nucleation sites for recrystallized grains. Moreover, because MEGs had a more favorable disposition to deformation than FGs, almost all slip bands were developed in MEGs at this stage, Figure 10a. Therefore, some GBs of MEGs were the main nucleation sites for new grains. However, the number of slip bands and dislocation density were insufficient to provide the adequate site of recrystallized grain nucleation and the adequate driving force of recrystallized grain growth at this stage; hence, MEGs were difficult to refine during solution treatment.

As single-pass rolling deformation continued, MEGs still tended to bear major plastic deformation and denser slip bands were progressively developed, causing more regions to experience dislocation pileups and higher dislocation density. During solution treatment, this boosted the recrystallization driving force and increased the nucleation site. At lower rolling reductions (such as 7%, 10%, and 13%), despite the formation of more slip bands, dislocation activity was still mainly concentrated on the primary slip systems. Moreover, even though multislip systems occurred due to uneven deformation in MEGs, the different regions in these abnormally coarse grains were still dominated by a single slip system. This indicated that slip bands in different regions of MEGs were still developed in one direction. Hence, under lower rolling reductions, the GBs of MEGs remain the main nucleation site for recrystallized grains. Although the recrystallization driving force was heightened and more nucleation sites developed at this rolling deformation stage, when refining the MEGs during solution treatment, the recrystallization was still mainly achieved through GB nucleation, which made it difficult to achieve satisfactory refinement effects for abnormally coarse elongated grains to eliminate mixed-grain structures after solution treatment.

At higher rolling reductions (such as 15% and 20%), multislip systems were activated, and slip bands along different directions were developed in the same regions of MEGs, Figure 10c. In addition to forming more slip bands and higher dislocation density, a large number of slip band intersections was also developed, which was beneficial for dislocation pileups and tangles and dislocation cell formation in the interior of the MEGs, as shown in Figure 10c. These dislocation structures can be effective nucleation sites for new grains within MEGs during recrystallization, promoting new grain formation [7]. After higher rolling reductions, therefore, significant GB nucleation and strong intragranular nucleation came about during solution treatment, contributing to the satisfying refinement of abnormally coarse elongated grains and eventually eliminating mixed-grain structures after solution treatment.

## 4. Conclusions

(1) In a heterogeneous hot-rolled microstructure, the millimeter-grade elongated grains (MEGs) possessed an extremely large size and a higher Schmid factor for slip compared to the fine grains (FGs). During subsequent single-pass deformation at 800℃, the regions with MEGs tended to undergo larger plastic deformation and accumulate at a higher dislocation density and deformation energy storage, contributing to new grain nucleation during final solution treatment and improving microstructure uniformity.

(2) At lower single-pass rolling reductions (7%, 10%, 13%), during final solution treatment, there were fewer nucleation sites for new grains, and the grain boundaries were the main nucleation site in MEGs, making it challenging to achieve satisfactory refinement effects for abnormally coarse elongated grains and then eliminate mixed-grain structures.

(3) At higher rolling reductions (15%, 20%), more nucleation sites and more uniformly distributed rolling deformations were developed in MEGs. As the reduction increased, the number of in-grain nucleation sites gradually exceeded that of grain boundary nucleation sites, and in-grain nucleation occurred preferentially during the final solution treatment. This was beneficial for promoting the refinement of new grains, lowering the difference in the size of new grains during recrystallization, achieving the satisfied refinement of abnormally coarse elongated grains, and eliminating mixed-grain structures.

## Figures and Tables

**Table 1:** Chemical composition (wt.%) of an ultra-purified ferritic stainless steel.

C	Si	Mn	Mo	V	Cu	N	Cr	Ni	Fe
0.032	0.51	10.53	4.83	0.032	0.07	0.21	12.19	11.82	Bal.

**Table 2:** The number of nucleation sites and preferential nucleation sites during recrystallization.

	7%	10%	15%	20%
GB nucleation	8	11	17	16
In-grain nucleation	1	2	3	17
Preferential nucleation site	GB	GB	GB	In-grain

## Data Availability

The original contributions presented in the study are included in the article/supplementary material, further inquiries can be directed to the corresponding author.

## Supplementary Materials

The following are available online at https://www.mdpi.com/article/10.3390/ma17102313/s1, Figure S1: Microstructure of the initial hot-rolled plate subjected to solution treatment.
