# Supplementary material for: Improvement in Grain Size Distribution Uniformity for Nuclear-Grade Austenitic Stainless Steel through Thermomechanical Treatment
Source: Materials (Basel). 2024 May 14;17(10):2313. doi: 10.3390/ma17102313 (PMC11122759; doi:10.3390/ma17102313)
Supplement: Supplementary file 1 [file materials-17-02313-s001.zip › materials-2901070-supplementary.pdf]

– Supporting Information –

# Improvement in Grain Size Distribution Uniformity for Nuclear-Grade Austenitic Stainless Steel through Thermomechanical Treatment

Yong Wang, Weiwei Xue, Zongxu Pang, Zichen Zhao, Zhuohua Liu, Chenyuan Liu, Fei Gao and Weijuan Li

**Figure S1**

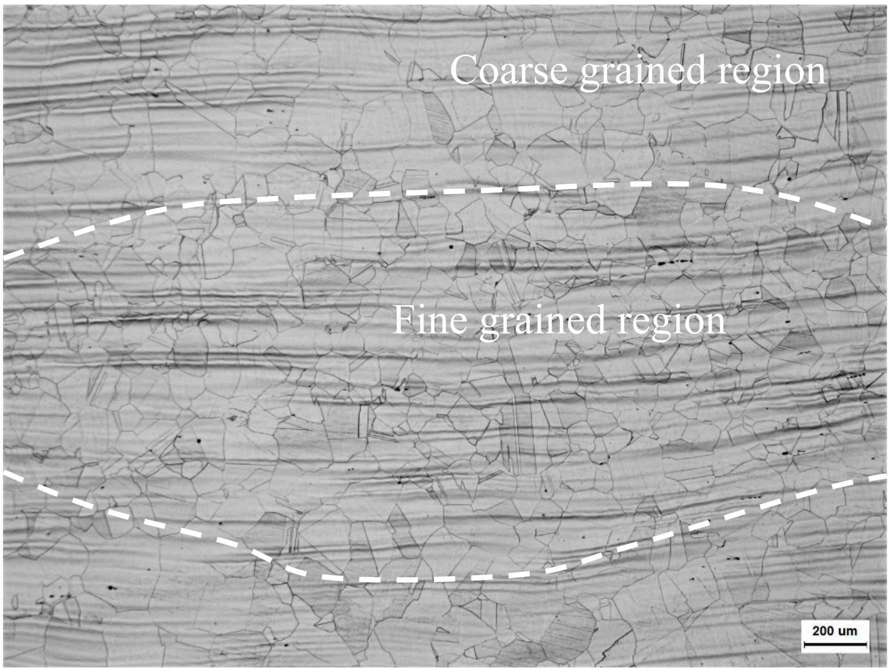


Figure S1 Microstructure of the initial hot rolled plate subjected to solution treatment
